# A multicenter comparison of quantification methods for antisense oligonucleotide-induced DMD exon 51 skipping in Duchenne muscular dystrophy cell cultures

**DOI:** 10.1371/journal.pone.0204485

**Published:** 2018-10-02

**Authors:** Monika Hiller, Maria Sofia Falzarano, Iker Garcia-Jimenez, Valentina Sardone, Ruurd C. Verheul, Linda Popplewell, Karen Anthony, Estibaliz Ruiz-Del-Yerro, Hana Osman, Jelle J. Goeman, Kamel Mamchaoui, George Dickson, Alessandra Ferlini, Francesco Muntoni, Annemieke Aartsma-Rus, Virginia Arechavala-Gomeza, Nicole A. Datson, Pietro Spitali

**Affiliations:** 1 Department of Human Genetics, Leiden University Medical Center, Leiden, The Netherlands; 2 UOL of Medical Genetics, University of Ferrara, Ferrara, Italy; 3 Neuromuscular Disorders Group, Biocruces Bizkaia Health Research Institute, Barakaldo, Spain; 4 Dubowitz Neuromuscular Centre, University College London Great Ormond Street Institute of Child Health, London, United Kingdom; 5 BioMarin Nederland B.V., Leiden, The Netherlands; 6 Centre of Biomedical Sciences, School of Biological Sciences, Royal Holloway University of London, London, United Kingdom; 7 Faculty of Health and Society, University of Northampton, Northampton, United Kingdom; 8 Department of Medical Statistics and Bioinformatics, Leiden University Medical Center, Leiden, The Netherlands; 9 INSERM, Institute of Myology, Center of Research in Myology, Sorbonne Universities, UPMC Univ Paris 6, Paris, France; 10 MRC Centre for Neuromuscular Diseases, University College London Great Ormond Street Institute of Child Health, London, United Kingdom; International Centre for Genetic Engineering and Biotechnology, ITALY

## Abstract

**Background:**

Duchenne muscular dystrophy is a lethal disease caused by lack of dystrophin. Skipping of exons adjacent to out-of-frame deletions has proven to restore dystrophin expression in Duchenne patients. Exon 51 has been the most studied target in both preclinical and clinical settings and the availability of standardized procedures to quantify exon skipping would be advantageous for the evaluation of preclinical and clinical data.

**Objective:**

To compare methods currently used to quantify antisense oligonucleotide–induced exon 51 skipping in the DMD transcript and to provide guidance about the method to use.

**Methods:**

Six laboratories shared blinded RNA samples from Duchenne patient-derived muscle cells treated with different amounts of exon 51 targeting antisense oligonucleotide. Exon 51 skipping levels were quantified using five different techniques: digital droplet PCR, single PCR assessed with Agilent bioanalyzer, nested PCR with agarose gel image analysis by either ImageJ or GeneTools software and quantitative real-time PCR.

**Results:**

Differences in mean exon skipping levels and dispersion around the mean were observed across the different techniques. Results obtained by digital droplet PCR were reproducible and showed the smallest dispersion. Exon skipping quantification with the other methods showed overestimation of exon skipping or high data variation.

**Conclusions:**

Our results suggest that digital droplet PCR was the most precise and quantitative method. The quantification of exon 51 skipping by Agilent bioanalyzer after a single round of PCR was the second-best choice with a 2.3-fold overestimation of exon 51 skipping levels compared to digital droplet PCR.

## Introduction

Duchenne muscular dystrophy (DMD) is a severe, X-linked neuromuscular childhood disorder caused by mutations in the *DMD* gene leading to lack of dystrophin [1]. Dystrophin has a critical function in providing structural stability to the muscle fibers and prevents their damage upon contraction. The disease occurs in ∼1:3,500 to 5,000 newborn males [2] and patients show progressive neuromuscular impairment from early childhood. Between the age of 6 and 12 years (mean 9.5 years), most boys lose ambulation and become wheelchair dependent [3], although corticosteroids administration, now standard of care for DMD, can slow down progression with mean age at loss of ambulation of approximately 13.5 years [4]. Additional disease milestones comprise loss of arm function, the need for assisted ventilation and premature death due to heart and respiratory failure [5,6]. The milder allelic form of the disease, known as Becker Muscular Dystrophy (BMD) is characterized by less severe symptoms, slower disease progression and longer life expectancy due to the production of shorter, partly functional dystrophin [7].

Antisense oligonucleotide (AON)-mediated exon skipping has been developed as an approach to facilitate the production of shorter, but partially functional dystrophin proteins, such as the ones found in BMD patients, in order to slow down disease progression for DMD patients. AONs are modified oligonucleotides that hybridize to a specific target exon at the pre-mRNA level and prevent exon inclusion by the splicing machinery [8–11]. Several AONs have been tested in clinical trials [12–15] and eteplirsen [16–18] is the first AON that recently received accelerated approval by the Food and Drug Administration in the United States [19,20]. This drug targets exon 51 of the DMD pre-mRNA and is applicable to 14% of DMD patients [21].

The choice of optimal biochemical outcome measures for preclinical and clinical trials for DMD is still debated. Researchers in this field are collaborating in order to test intra- and inter-variability for a set of methods aimed at quantifying the levels of dystrophin protein restoration [22–24]. In the case of clinical trials, a coordinated effort has been made to validate dystrophin quantification methods to study the restoration of dystrophin protein [25,26]. The biochemical outcome measures study group (BOM-SG), a group of several institutions interested in the development of therapeutic AONs, compared protein levels by quantitative immunohistochemistry and Western blotting with the aim to provide guidance on how to correctly quantify dystrophin in muscle biopsies. The relevance of this effort has been demonstrated as eteplirsen was recently approved on the basis of dystrophin protein restoration [20].

Current preclinical development of new AONs (e.g. to optimize AONs for exons not yet in clinical development or the next generation AONs for exons that are in clinical development) is performed on the basis of their exon skipping activity at RNA level. However, no standardized protocol is available to determine exon skipping levels, making the comparison of exon skipping efficiency among clinical trials and also in preclinical studies difficult. The preclinical development of AONs relies on methods that have yet to be evaluated and validated. As part of the efforts of the BOM-SG, we tested different methods available to quantify exon skipping levels in cultured cells with the aim to provide guidance on exon skipping quantification.

## Materials and methods

### Cell culturing, AON transfection and RNA isolation

Two transfection experiments were performed to provide the participating groups with RNA aliquots to quantify exon skipping levels. Since the analysis of the first transfection experiment yielded low exon skipping levels a second transfection experiment was performed using a slightly different protocol to obtain higher exon skipping levels. The detailed description of the cell culturing, transfection and RNA isolation can be found in the Supporting Information. Briefly, immortalized human patient myoblasts with a deletion of *DMD* exon 52 (cell line 1531) and *DMD* exons 48–50 (cell line 8036), both kindly provided by Vincent Mouly, Institute de Myologie, Paris, France [27,28], were cultured and differentiated into myotubes. Cells were transfected with AON h51AON2 targeting exon 51 (S1 Table) [29], an antisense oligonucleotide with 2’-O-methyl-modified bases, a phosphorothioate backbone and a 5’ fluorescent tag to check transfection efficiency, kindly provided by BioMarin. AONs were transfected at (50), 200 and 400 nM to obtain (low), medium and high exon skipping levels. Lipofectamine-only treated cells, “untreated cells” served as a negative control. RNA was isolated 48h after transfection, RNA concentration and purity were determined, and exon skipping levels were confirmed before identical aliquots were shipped to participant laboratories (see S1 Supplementary information for the detailed method description).

### Distribution of RNA aliquots and protocols

RNA samples were blinded and sent to the participating partners to perform exon skipping quantification. The laboratories received the following information: i) which three biological replicates belonged together, ii) the cell line each sample belonged to iii) the AON concentrations transfected (without disclosing this for the individual samples, since the laboratories had to assess the concentrations (blinded samples)).

Our aim was to perform the same exon skipping quantification protocols in several laboratories to allow a comparison per technique and among techniques. Therefore, comprehensive protocols containing all information to allow replication of each step (e.g. step by step descriptions, reagents with lot numbers, sequences etc.) were shared between the groups. Depending on the availability of the required equipment, not all groups performed all quantification methods (Table 1). Protocols were strictly followed to reduce bias.

**Table 1:** Overview of the performed technologies.

Performing laboratory	1^st^ transfection experiment (Lab 1)					2^nd^ transfection experiment (Lab 3)	
	ddPCR method	Bioanalyzer method	Densitometry_ ImageJ method	Densitometry_ GeneTools method	qPCR method	ddPCR method	Bioanalyzer method
**Lab 1**							✓
**Lab 2**	✓					✓	
**Lab 3**	✓	✓	✓		✓	✓	✓
**Lab 4**		✓	✓	✓			✓
**Lab 5**			✓		✓		✓
**Lab 6**		✓	✓		✓		✓

Overview of the technologies performed by the different laboratories. Four protocols were included for the 1^st^ transfection experiment and the two most promising technologies (ddPCR and single round RT-PCR combined with bioanalyzer quantification) were replicated with samples of the 2^nd^ transfection experiment. ddPCR = digital droplet PCR and qPCR = quantitative real-time PCR.

### Protocols to quantify exon skipping levels

#### Protocol: Digital droplet PCR (ddPCR) (ddPCR method)

cDNA synthesis. ddPCR was performed as described in Verheul et al. [30]. cDNA was generated from 750 ng of RNA with Transcriptor Reverse Tanscriptase (#03531287001, Roche), random hexamer primers (#11034731001, Sigma-Aldrich), RNasin ribonuclease inhibitor (#N2115, Promega) and dNTPs (#11581295001, Sigma-Aldrich) in a total volume of 20 μl according to the manufacturer’s instructions. As a negative control, one reaction without reverse transcriptase was included.

ddPCR. Taqman assays from Thermo Fisher Scientific were ordered as 20-fold concentrations to quantify dystrophin cDNA products with or without exon 51. Primers and probes for Δ48–50 and Δ52 cell lines were identical to those described by Verheul et al. [30] (for sequences see S1 Table). The probes we used bind to the exon-exon junction (EEJ) of the transcripts with and without skipping of exon 51; for the skipped assay and non-skipped assay of Δ48–50 samples the probes bind to EEJ 47/52 and EEJ 51/52 respectively, and for the skipped assay and non-skipped assay of Δ52 samples the probes bind to EEJ 50/53 and EEJ 51/53 respectively. With this design the probe for the skipped assay is specific only for transcripts which lack exon 51 completely.

Separate reactions to detect the skipped and non-skipped products were prepared in a semi skirted 96-well plate (#0030 128.591, Eppendorf), containing 11 μl of 2x ddPCR Supermix for Taqman assays (#1863023, Bio-Rad), 1.1 μl of 20x Taqman assay, 2.2 μl of undiluted cDNA and 7.7 μl of DNase/RNase-free H_2_O per reaction. Taqman assays ‘Skip_del48-50’ and ‘Non-skip_del48-50’ were used for 8036 cells and ‘Skip_del52’ and ‘Non-skip_del52’ for 1531 cells to quantify skipped and non-skipped fragments respectively. One reaction without cDNA was included as negative control. The plate was spun down to collect contents at the bottom of the wells before droplet generation.

Droplets were generated with the automated droplet generator QX200 system (#1864100, Bio-Rad) according to the provided supplier instructions, using ddPCR cartridges (#1864108, Bio-Rad) and 70 μl droplet generation oil for probes (#1863005, Bio-Rad) per 20 μl of sample. After finishing droplet generation, the plate was sealed with tin foil (#1814040, Bio-Rad) for 4 seconds at 170°C using the PX1 PCR plate sealer (#1814000, Bio-Rad).

The samples were amplified in a T100 thermal cycler (Bio-Rad) using the following program: 10 min at 95°C, 40 cycles of 30 sec at 95°C and 1 min at 60°C, 10 min at 98°C and held at 8°C. The heated lid was set to 105°C and the reaction volume to 40 μl. The plate containing the amplified droplets was transferred to the droplet QX200 reader (#1864100, Bio-Rad) to count positive (with cDNA) and negative droplets (without cDNA).

Exon skipping quantification. Data were analysed with QuantaSoft software, version 1.7 (#1864011, Bio-Rad), and could be visualized as 1-D or 2-D plots to show the separation between negative and positive droplets. The fluorescent amplitude threshold was set manually to discriminate between positive and negative droplets. The absolute concentration was represented in copies/μl sample mix and the concentrations of the skipped and the non-skipped assay were used to calculate exon skipping percentages according to the formula: Exon skipping % = (skipped copies/μl)/(skipped copies/μl + non-skipped copies/μl) x 100% [30]. This formula can be applied directly when the same volumes of cDNA were added to the skipped and non-skipped ddPCR reactions (as it was the case in our experiment). If the volumes differ, the target concentration present in the cDNA sample can be calculated with the formula: Target concentration cDNA (copies/μl) = absolute concentration (copies/μl) * total volume PCR reaction (μl)/volume cDNA (μl). In this case the target concentration of the cDNA should be used to calculate the exon skipping percentage.

#### Protocol: Single PCR combined with Agilent bioanalyzer analysis (bioanalyzer method)

cDNA synthesis. cDNA synthesis was performed with 300 ng of RNA with the High-Capacity cDNA Reverse Transcription Kit (#4368814, Thermo Fisher Scientific) in a 20 μl reaction volume according to the manufacturer’s instructions. RNaseOUT (#10777019, Thermo Fisher Scientific) was used in a final concentration of 2 U/μl. One reaction was included without transcriptase as a negative control.

Single round PCR. The PCR reactions contained final concentrations of 1 x buffer (#10966–034, Thermo Fisher Scientific), 0.2 mM dNTPs (#4030, Takara), 0.4 μM forward primer (Eurogentec), 0.4 μM reverse primer (Eurogentec) (for sequences see S1 Table), 1.5 mM MgCl_2_ (#10966–034, Thermo Fisher Scientific), 0.04 U/μl Platinum Taq DNA polymerase (#10966–034, Thermo Fisher Scientific) and 1 μl of cDNA in a total volume of 25 μl. The PCR was run for 2 min at 94°C, 30 cycles of 45 sec at 94°C, 45 sec at 60°C and 80 sec at 72°C, then 5 min at 72°C and cooled down to room temperature. For 8036 cells (*DMD*Δ48–50) the expected fragment sizes using primers h45F and h52R (S1 Table) were 539 bp (non-skipped) and 306 bp (skipped) products. For 1531 cells (*DMD*Δ52) the expected fragment sizes were 800 bp (non-skipped) and 567 bp (skipped) with primer pair h49F and h53R1 (S1 Table).

Exon skipping quantification. PCR products were quantified with the Agilent high sensitivity DNA kit (#5067–4626, Agilent) on an Agilent 2100 Bioanalyzer according to the manufacturer’s instructions. The exon skipping percentages were calculated as the ratio of skipped transcripts and total transcripts according to the formula: Exon skipping % = (molarity skipped transcripts)/(molarity skipped transcripts + molarity non-skipped transcripts) x 100%.

#### Protocol: Nested PCR combined with agarose gel electrophoresis and densitometry analysis by ImageJ software (densitometry_ImageJ method)

cDNA synthesis. cDNA synthesis was performed with 400 ng of RNA with Transcriptor Reverse Transcriptase (#03531287001, Roche), using random hexamers (#SO142, Thermo Fisher Scientific), RNAsin ribonuclease inhibitor (#N2515, Promega) and dNTPs (#10297018, Thermo Fisher Scientific) in a total volume of 20 μl according to the manufacturer’s instructions, with minor adaptations: i) a lower concentration of random hexamers (final concentration 2 ng/μl) was used and ii) cDNA was prepared at 42°C. For one reaction, no transcriptase was included as a negative control.

Nested PCR. For the amplification 25 μl PCR reactions containing 1 x Supertaq PCR buffer (# TPRB, Sphaero Q), 0.2 mM of each dNTP (#10297018, Thermo Fisher Scientific), 0.4 μM forward primer (h47F1, Eurogentec, see S1 Table), 0.4 μM reverse primer (h54R, Eurogentec, see S1 Table), 0.025 U/μl Taq DNA polymerase (#11146165001, Roche) and 3 μl undiluted cDNA were prepared. Samples were run in a PCR machine for 5 min at 94°C, 20 cycles of 40 sec at 94°C, 40 sec at 60°C and 80 sec at 72°C, then 7 min at 72°C and cooled down to room temperature.

For the nested PCR, 1.5 μl of the first PCR product was mixed with 48.5 μl PCR master mix with final concentrations of 1 x Supertaq PCR buffer (# TPRB, Sphaero Q), 0.2 mM dNTPs (#10297018, Thermo Fisher Scientific), 0.4 μM forward primer (h47F2, Eurogentec, see S1 Table), 0.4 μM reverse primer (h53R2, Eurogentec, see S1 Table) and 0.025 U/μl Taq DNA polymerase (#11146165001, Roche). The 50 μl PCR reactions were run for 5 min at 94°C, 32 cycles of 40 sec at 94°C, 40 sec at 60°C and 60 sec at 72°C, then 7 min at 72°C and cooled down to room temperature. The PCR products were visualised by agarose gel electrophoresis after samples were mixed with 5 μl orange G (10-fold stock solution: 2 g/L Orange G, 30 v/v% glycerol). Five μl were loaded onto a 2% TBE-agarose gel containing 0.3 μg/ml ethidium bromide. The gel was run for 1 h at 100 V in 1xTBE buffer (10.8 g/L of Tris base, 5.5 g/L of Boric acid, 2 mM EDTA). Expected PCR fragment sizes were 475 bp (non-skipped) and 242 bp (skipped) for Δ48–50 cells and 754 bp (non-skipped) and 521 bp (skipped) for Δ52 cells and were checked with a 100 bp ladder (#SM0322, Thermo Fisher Scientific).

Exon skipping quantification. All pictures of the agarose gels obtained at the different laboratories were collected by one laboratory and analysed by one researcher with the ImageJ software. The peak area of the skipped and the non-skipped fragments were determined and corrected for their amplicon sizes. Exon skipping percentages were calculated as exon skipping % = corrected peak area of the skipped fragment/(corrected peak area of the skipped fragment + corrected peak area of the non-skipped fragment) x 100%.

#### Protocol: Nested one step RT-PCR combined with agarose gel electrophoresis and densitometry analysis by GeneTools software (densitometry_GeneTools method)

cDNA synthesis and first round PCR. The cDNA synthesis and first round of PCR was prepared with the GeneScript RT PCR system from Quantig (#GS003) to transcribe single-stranded RNA into double-stranded DNA followed by amplification. Four hundred (400) μg of RNA was used for a 25 μl reaction volume containing 200 μM dNTPs, 1X reaction buffer, 300 nM forward primer (h47F2, S1 Table), 300 nM reverse primer (h54R, S1 Table), and 1.25 U *Accurase*/MMLV reverse transcriptase/RNase inhibitor. The reactions were run for 30 min at 45°C, 5 min at 92°C followed by 20 cycles of 30 sec at 92°C, 30 sec at 60°C and 45 sec at 68°C, finalized for 10 min at 68°C and held at 4°C. One sample without enzyme was used as a negative control.

Nested PCR. The PCR was prepared with 2 x PCR mastermix from Quantig (#PCRM002RD), 2 μl of PCR product from the first amplification, 200 μM dNTPs, 300 nM forward primer (h47F1, S1 Table) and 300 nM reverse primer (h53R2, S1 Table) in a reaction volume of 25 μl. The cycling conditions for the PCR were 2 min at 92°C followed by 30 cycles of 30 sec at 92°C, 30 sec at 600°C and 1 min at 68°C, followed by 10 min at 68°C and then held at 4°C. Ten (10) μl of the PCR products were loaded on a 2% TBE agarose gel with 1X SYBR Safe Gel Stain (#S33102, Invitrogen) and run against a 100 bp ladder (#BIO-33056, Bioline) at 100V for 45 minutes.

Exon skipping quantification. The peak area was determined with the GeneTools imaging analysis software from Syngene and was corrected for PCR fragment sizes (same fragment sizes as with ImageJ analysis). The following formula was used for the exon skipping quantification: Exon skipping % = corrected peak area of the skipped fragment/(corrected peak area of the skipped fragment + corrected peak area of the non-skipped fragment) x 100%.

#### Protocol: Quantitative real-time PCR (qPCR) (qPCR method)

cDNA synthesis. This protocol was replicated as previously described by Anthony et al. [31]. cDNA synthesis was performed with SuperScript III First-Strand Synthesis SuperMix (#18080400, Thermo Fisher Scientific). Per reaction, 500 ng of RNA, 1 μl of annealing buffer and 1 μl of random hexamer primers (50 ng/μl) were mixed together and made up to 8 μl with RNase/DNase free water. Reactions were incubated in a thermal cycler at 65°C for 5 min and then immediately placed on ice for at least 1 min. After adding 10 μl of 2X First-Strand Reaction Mix and 2 μl Superscript III/RNase OUT Enzyme Mix, reactions were run for 10 min at 25°C, 50 min at 50°C and 5 min at 85°C. One reaction without transcriptase was used as negative control (-RT reaction). Samples were cooled on ice and diluted 5-fold in RNase/DNase free water for the cDNA pre-amplification.

Pre-amplification PCR. The pre-amplification step was performed using the TaqMan PreAmp Master Mix (#4391128, Thermo Fisher Scientific), according to the manufacturer’s instructions. Taqman assays (see S1 Table) were obtained from Thermo Fisher Scientific (20X concentrations) and were 100-fold diluted by adding 1 μl of the skipped assay (Skip_exon47-52 or Skip_exon50-53) and 1 μl of the non-skipped assay (Non-skip_ exon51-52 or Non-skip_exon51-53) to 98 μl RNase/DNase free water for each cell line. The reaction volume was scaled down to 25 μl containing 12.5 μl PreAmp Master Mix, 6.25 μl mixed assays (skipped and non-skipped with final concentration 0.05X of each), 5 μl of 5-fold diluted cDNA and 1.25 μl RNase/DNase free water. Reactions were run in a thermocycler for 10 min at 95°C followed by 14 cycles of 95°C for 15 sec and 60°C for 4 min. The pre-amplified cDNA was 5-fold diluted to be used as template for performing the qPCR assays.

qPCR. qPCRs were performed on a Roche LightCycler 480 (lab 3), StepOnePlus real-time PCR system from Thermo Fisher Scientific (lab 5) or 7900HT Fast Real-Time PCR system from Applied Biosystems (lab 6). For the amplification of the skipped and non-skipped transcripts, a mastermix for each Taqman probe was prepared, containing 12.5 μl TaqMan Universal PCR master mix (#4369016, Thermo Fisher Scientific), 1.25 μl probe (Non-skip_exon51-52, Skip_exon47-52, Non-skip_exon51-53 or Skip_exon50-53 (see S1 Table)), 6.25 μl 5-fold diluted cDNA from the pre-amplification and 5 μl DNase/RNase free water. As negative controls, one reaction without template and one reaction with the–RT cDNA from the cDNA synthesis were included. Twenty-five μl were pipetted into the wells, and the qPCR plate was sealed and spun down for 2 min at 1000 rpm. The program for the qPCR machines was as follows: 2 min at 50°C, 10 min at 95°C, 40 cycles of 15 sec at 95°C and 1 min at 60°C, and held at 37°C.

Exon skipping quantification. Data were analysed with LinReg [32,33] to calculate the PCR efficiency per individual well. Starting concentrations (N_0_) and Ct values were determined and used to calculate exon skipping percentages with the following formula: N_0_ skipped transcripts / (N_0_ skipped transcripts + N_0_ non-skipped transcripts) x 100 [31].

### Statistics

Data were analysed with R using R packages ‘dplyr’, ‘tidyr’, and ‘dglm’ (R version 3.3.2). The ddPCR results obtained by lab 2, a method to absolutely quantify nucleic acid target sequences with a high-precision, were used as a reference, since this laboratory had the most experience with this technique. To test whether the results obtained with other methods differed from the ddPCR results, a double generalized linear model was applied, which allowed to simultaneously model the mean and the dispersion of not normally distributed data.

First, exon skipping levels were log-transformed. To avoid an error in the transformation for exon skipping levels of 0% in the untreated samples (0 nM AON concentration), all exon skipping values were summed before the log transformation with the arbitrary number of 1. For further analysis, untreated samples were excluded from the analysis to avoid a dispersion of zero.

We tested the effects of the interaction between AON concentration and sample set (meaning from which transfection and cell line the sample was obtained), and the interaction between protocol and performing laboratory. We determined the mean of the exon skipping levels and the dispersion around the mean. For the mean submodel, both interaction terms were used in the argument, and for the dispersion submodel, only the interaction between protocol and performing laboratory was included. *P*-values for the mean and dispersion were calculated, and *P*-values less than 0.05 were considered significant. Significant *P*-values indicated that a particular quantification method was less reliable.

## Results

### Experimental design and sample identification

**Fig 1:**
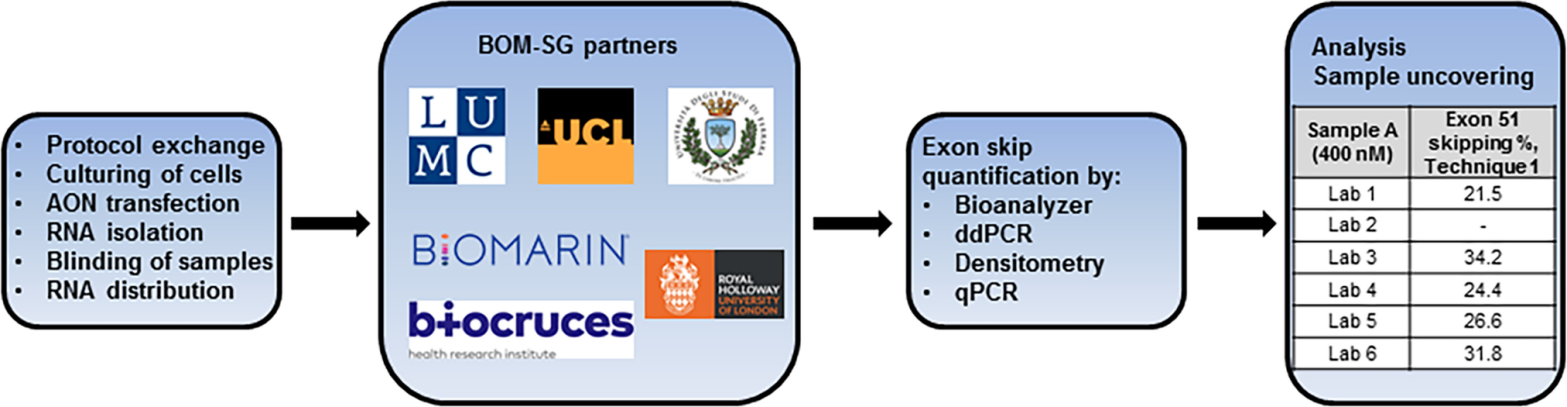
Flow diagram of the study design. Two transfection experiments and exon skipping quantification were performed. AON = antisense oligonucleotide, BOM-SG = biochemical outcome measures group, ddPCR = digital droplet PCR and qPCR = quantitative real-time PCR.

Two DMD muscle cell lines (*DMD*Δ48–50 (8036 cells) and *DMD*Δ52 (1531 cells)) were transfected with an AON able to skip exon 51. Two transfection experiments and analysis were performed, since the first transfection experiment yielded low exon skipping levels. Blinded RNA aliquots were distributed to all participating groups. All methods were tested in samples obtained during the first transfection experiment, while only the methods that resulted into the most robust results from the first set of experiments (ddPCR and the bioanalyzer method) were tested in samples obtained during the second transfection experiment. A flow diagram of the study design is shown in Fig 1 and an overview of the type of quantification protocol used by each laboratory is given in Table 1.

Zero nM, (50 nM), 200 nM and 400 nM AON concentrations were used as to induce no, (low), medium and high levels of exon skipping, respectively. For the first transfection experiment, in which all methods were tested, laboratories were able to correctly assign AON concentrations to the blinded samples in 96.9% of the cases for the untreated samples, 93.7% of the cases for the low skipping samples, 75.0% of the cases for the medium skipping samples and 75.0% of the cases for the high skipping samples. For RNA samples of the second transfection, laboratories assessed all AON concentrations correctly.

After collecting all data, samples were un-blinded and results were compared and analyzed; all exon skipping results are listed in S2 Table. Examples of raw data for determining exon skipping levels by ddPCR, bioanalyzer and densitometry are given in S1 Fig.

### Comparison of methods

**Fig 2:**
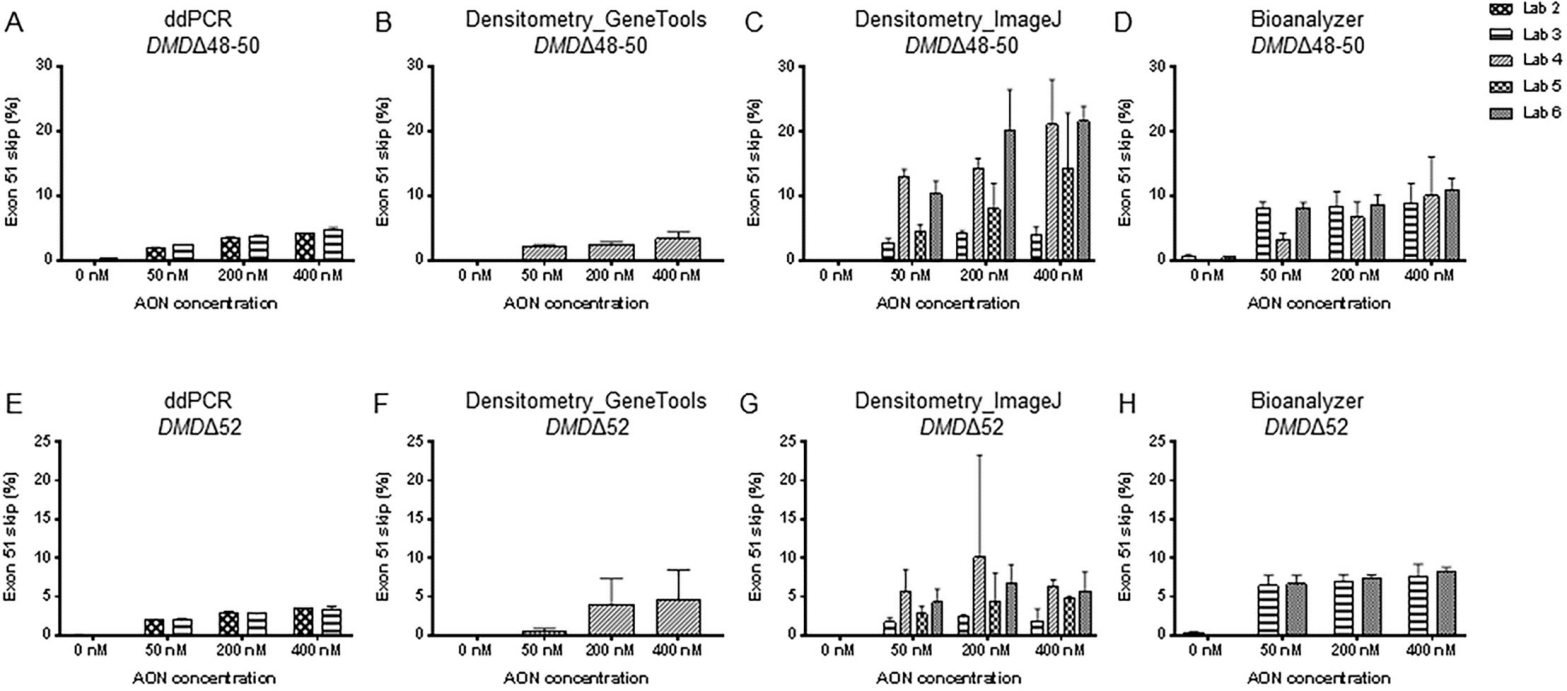
Bar graphs showing exon skipping levels of *DMD*Δ48–50 (A-D) and *DMD*Δ52 (E-H) cells after transfection with AON h51AON2 to skip exon 51 (1^st^ transfection experiment). Four different protocols were tested. Error bars represent standard deviation.

**Fig 3:**
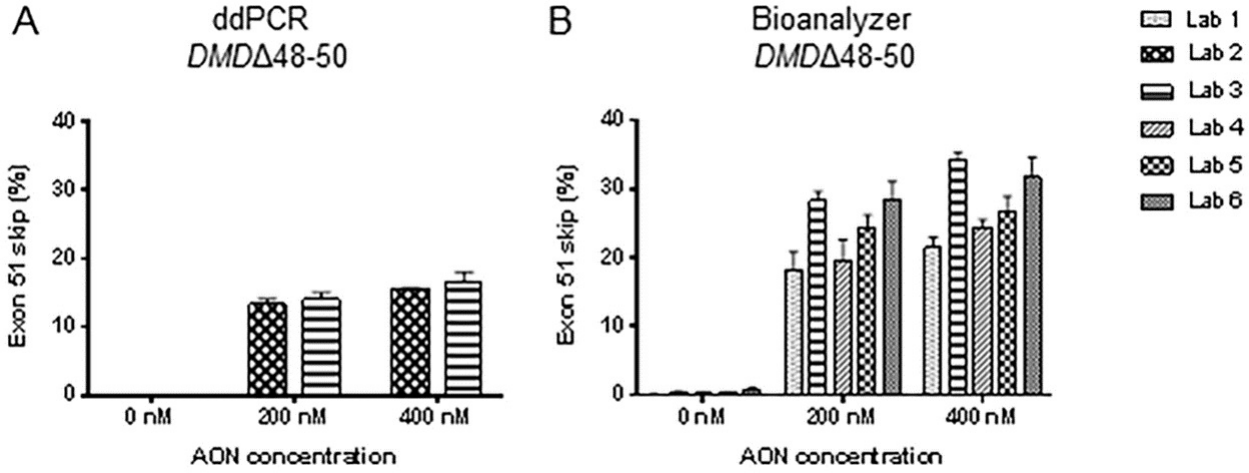
Bar graphs showing exon skipping levels of *DMD*Δ48–50 (A-B) cells after transfection with AON h51AON2 to skip exon 51 (2^nd^ transfection experiment). Two different protocols were tested. Error bars represent standard deviation.

**Fig 4:**
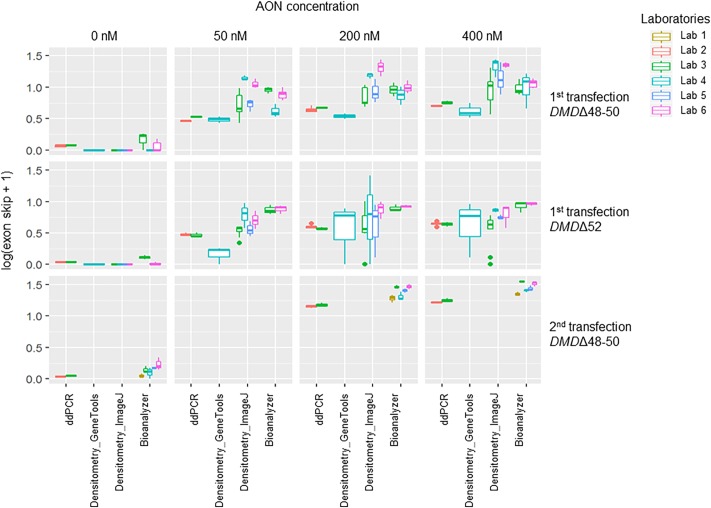
Boxplots of log transformed exon skipping levels (y-axis) are shown per technology (x-axis). The figure is divided into three horizontal panels representing the different sets of samples and four vertical panels illustrating the AON concentrations used for the transfections. Exon skipping levels of the different labs are shown in colours (lab 1 = yellow, lab 2 = red, lab 3 = green, lab 4 = turquoise, lab 5 = blue and lab 6 = pink). Outliers are represented by coloured dots.

Bar graphs reporting exon skipping data for all samples are presented in Figs 2 and 3 for the first and second transfection experiment, respectively. All data were combined in one statistical model (except for qPCR exon skipping levels, see next paragraph) and boxplots of log transformed exon skipping levels are shown in Fig 4. ddPCR data showed little dispersion for both performing labs (labs 2 and 3, Table 2); the deviation of exon skipping levels obtained by the two labs was very small with less than 0.4% difference on average. However, a statistically significant difference was observed between the two labs due to high level of precision, which was somewhat affected by upstream sample preparation steps such as cDNA synthesis that lead to a significant deviation in the mean exon skipping percentage (P<0.01, Table 2). No significant difference was detected between the densitometry_GeneTools method and the results obtained by ddPCR due to the high variation seen in the image based quantification as depicted by the high reported dispersion (Fig 2 and Table 2). Compared to the ddPCR data, both densitometry_ImageJ method and bioanalyzer method overestimated the exon skipping percentage, by a mean factor of 2.6 (densitometry_ImageJ method) and 2.3 (bioanalyzer method) (P<0.01 and P<1E-06 for all labs, respectively, Table 2). The dispersion of the densitometry_ImageJ method was higher than the bioanalyzer method. In the untreated samples, spontaneous exon skipping of exon 51 was detected by ddPCR and bioanalyzer methods, while other technologies were not able to detect the occurrence of this phenomenon due to their lower sensitivity (Fig 2 and Table 2).

**Table 2:** *P*-values obtained for the double generalized linear model.

	ddPCR		Bioanalyzer		Densitometry_ImageJ		Densitometry_GeneTools	
	mean	dispersion	mean	dispersion	mean	dispersion	mean	dispersion
**Lab 1**		-	1.89E-11	**3.06E-01**		-		-
**Lab 3**	6.85E-03	**8.16E-01**	1.32E-48	2.34E-09	1.61E-03	5.96E-48		-
**Lab 4**		-	3.53E-07	2.45E-13	2.02E-07	1.89E-32	**1.32E-01**	1.90E-27
**Lab 5**		-	8.37E-40	**9.34E-01**	5.37E-04	3.25E-25		-
**Lab 6**		-	7.15E-95	1.41E-06	1.89E-13	5.82E-25		-

*P*-values obtained for the double generalized linear model for the mean and the dispersion considering different AON concentrations and sample sets. The ddPCR performed by lab 2 was used as reference. *P*-values less than 0.05 were considered significant. Significant *P*-values indicate a less reliable quantification method due to a higher variation to the reference, not significant *P*-values indicate a reliable method and are shown in bold.

Exon skipping levels quantified by qPCR were not in accordance with the results obtained by other methodologies and were thus excluded from the statistical analysis. Exon skipping percentages obtained by qPCR are shown in S2 Fig. Values measured by qPCR were much higher compared to the reference values (ddPCR performed by lab 2). We assessed a 174-fold increase in 8036 untreated cells, a 33-fold increase for 50 nM of AON, a 15-fold for 200 nM of AON and 13-fold for 400 nM of AON, compared to the reference values. For 1531 cells the levels were also higher compared to the reference values, but less extreme (10-fold in untreated cells, 7-fold for 50 nM of AON, 4-fold for 200 nM of AON and 3-fold for 400 nM of AON).

Three techniques (ddPCR, densitometry_ImageJ method and bioanalyzer method) were also replicated 2 or 3 times by one operator to examine the intra-laboratory variability (S3 Fig). The highest intra-operator variability was detected with the densitometry_ImageJ method. Less intra-operator variability was obtained for the other two techniques, ddPCR and bioanalyzer methods.

## Discussion

In this study, we quantified exon 51 skipping in DMD myotube cultures with different technologies, to test the precision of the most widely used techniques and to provide guidance over the method(s) to use.

In our study, technologies such as single PCR assessed with Agilent bioanalyzer, nested PCR with densitometry image analysis by either GeneTools or ImageJ software, and qPCR were compared to the method we selected as reference, ddPCR. We performed a first transfection experiment and tested the different technologies previously listed. Unfortunately, the exon skipping levels obtained were quite low for all AON treatments of the first transfection experiment, probably due to a suboptimal transfection. While this was not anticipated, it did allow us to work with low exon skipping levels, which might be comparable to the ones obtained in clinical trials. For the second transfection experiment higher exon skipping levels were achieved with a slightly different transfection protocol.

Each technology showed advantages and disadvantages. The ddPCR is a quantitative method which detects single nucleic acid molecules compartmentalized in droplets with a high sensitivity and precision even at low template copy numbers. When the amplified product of this endpoint PCR is present in the droplet, a fluorescent signal is induced which allows absolute quantification of all positive droplets. This is the most precise method available to determine exon skipping levels, and it was shown not to overestimate exon skip levels [30]. The workflow and analysis of the ddPCR protocol are very simple. Attention has to be paid in the assay design which was done previously by Verheul et al. [30]. Due to the fact that we used the same volumes of undiluted cDNA in the skipped and non-skipped PCR reactions, exon skipping levels could be calculated directly with the concentrations given by the machine. We were able to show that exon skipping levels obtained with this method were comparable across two different laboratories. Very likely because the high precision causes a very narrow data distribution, a significant difference was observed between the two labs that replicated the method (5.8% exon skipping (lab 2) vs. 6.2% exon skipping (lab 3) on average).

A disadvantage of this method is represented by the high costs associated to the equipment and reagents, which limits its availability. Reduction of costs and increase of performance could be achieved by using two different fluorescent dyes for the skipped and non-skipped probes, enabling to pool two reactions in a single tube as well as a high-throughput sample preparation in an automated system.

In clinical trials there should be a control for the amount of myogenic content of muscle fibers in the samples, since due to the pathology muscle biopsies from DMD patients generally will contain a lot of adipose tissue. The myogenic content in a sample can be checked with an additional assay quantifying the expression of muscle, adipose and fibrotic tissues specific genes. This would enable to estimate to which extent each tissue contributes to the total amount of RNA purified from the biopsy. To get an impression of full transcript levels, multiple assays with primers and probes binding along the dystrophin transcripts can be included as well to improve the ddPCR workflow.

The bioanalyzer method resulted in a 2.3-fold overestimation of exon skipping levels compared to the ddPCR method. A reason could be that amplification of the shorter skipped fragments is more efficient compared to longer non-skipped fragments during PCR. However, the low inter-operator variability, the low data dispersion and the relatively lower performing costs, represent advantages in favor of this technique.

The densitometry_ImageJ method uses a nested PCR to increase sensitivity. Our data showed that this approach led to a 2.6-fold exon skipping overestimation. Factors that influenced this outcome were gel image acquisition and peaks definition within ImageJ. In fact, when a single operator processed all the acquired images, exon skipping levels showed less variation compared to when different operators were involved (data not shown). Even though more amplification cycles were performed, the sensitivity of the method was not improved since spontaneous exon skipping in untreated samples seen in ddPCR and bioanalyzer methods could not be detected. In our opinion, the ease of the protocol, the fact that no specific instruments are needed and the limited costs do not outweigh against the low precision and low sensitivity of the assay.

The densitometry_GeneTools method was tested by one single laboratory, so it is not possible to draw conclusions on the overall reproducibility of the method. However the high dispersion observed in the data obtained by lab 4 suggest that this method is unlikely to be able to reliably quantify exon 51 skipping.

Quantification of exon skipping by qPCR led to large variability among different laboratories, with some laboratories largely overestimating exon skipping levels. This is likely due to the high number of steps involved in this protocol, which includes a pre-amplification step, and the difficulties in standardization across laboratories. In addition, this method was optimized and validated using muscle tissue [31]. While a qPCR method would largely benefit the community to reduce the costs derived from the ddPCR approach, further method optimization is required to consider this method a good ddPCR surrogate for exon skipping quantification.

Based on the data obtained, we suggest that the ddPCR protocol should be used to determine exon 51 skipping levels. ddPCR was the most precise and quantitative method. Quantification of a single round PCR using an Agilent bioanalyzer represents a fair alternative, considering that it is the method that less overestimates exon skipping by an acceptable factor of 2.3-fold compared to ddPCR. The use of a standardized protocol to quantify exon skipping levels would enable the comparison of novel interventional drugs with the previous generation of drugs. Exon skipping is an important readout and combining exon skipping quantification with the analysis of dystrophin protein restoration by e.g. western blot analysis [25] would be more informative to better estimate the size of treatment effects on RNA and protein levels and properly power clinical studies.

## Supporting information

S1 Fig **Examples of raw data to determine exon skipping levels by ddPCR (A), bioanalyzer (B) and densitometry (C) of Δ48–50 cells treated with an AON to skip exon 51.** A. The 1D amplitude plot shows positive (blue) and negative dots (grey) for the skipped and the non-skipped assays. B. Results of the electrophoresis run of the high sensitivity DNA assay showing the non-skipped fragment at 539 bp and the skipped fragment at 306 bp. C. The agarose gel shows the two fragments after electrophoresis; the non-skipped fragments at 475 bp and the skipped fragment at 242 bp.
(TIF)

S2 Fig **Intra-laboratory variation of exon 51 skipping levels in *DMD*Δ48–50 (A-C) and *DMD*Δ52 cells (D-E).** Three different protocols were repeated by the same operator (n = 2/3). Error bars represent standard deviation.
(TIF)

S3 Fig Exon skipping levels of *DMD*Δ48–50 and *DMD*Δ52 cells obtained by qPCR (1^st^ transfection experiment). Error bars represent standard deviation.
(TIF)

S1 Table Sequences of primers, probes and AON used. FAM = 6-carboxyfluorescein label, 2’OMePS = 2'-O-methyl-modified bases on a phosphorothioate backbone.
(DOCX)

S2 Table Exon 51 skipping percentages obtained by the different laboratories for the different technologies. Three biological replicates of each sample were measured. SD = standard deviation.
(DOCX)

S1 Supplementary information Detailed description of cell culture conditions, transfection and RNA purification. (DOCX)
