# Supplementary figures and images for: A multicenter comparison of quantification methods for antisense oligonucleotide-induced DMD exon 51 skipping in Duchenne muscular dystrophy cell cultures
Source: PLoS One. 2018 Oct 2;13(10):e0204485. doi: 10.1371/journal.pone.0204485 (PMC6168132; doi:10.1371/journal.pone.0204485)

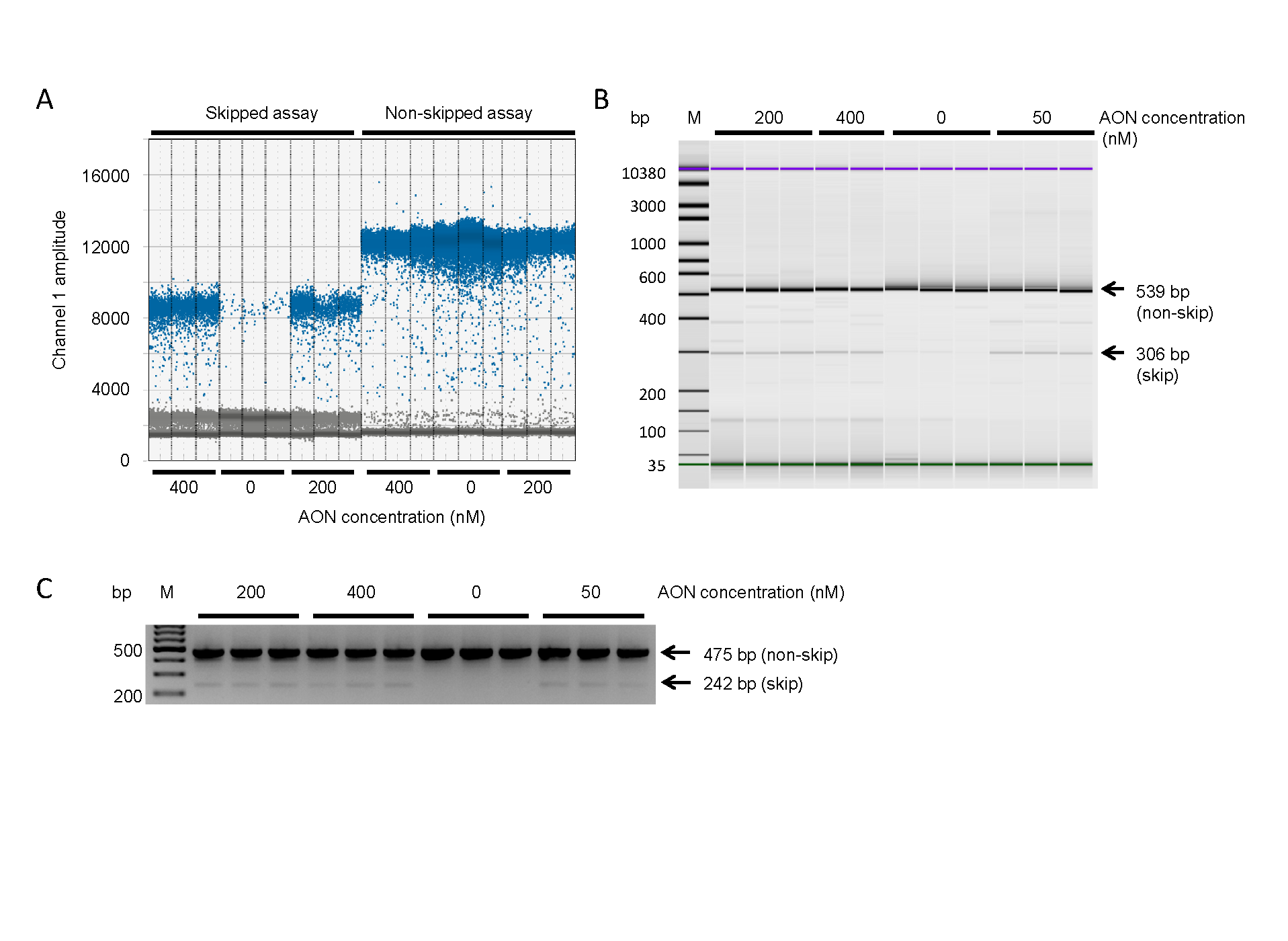

Supplement: S1 Fig — Examples of raw data to determine exon skipping levels by ddPCR (A), bioanalyzer (B) and densitometry (C) of Δ48–50 cells treated with an AON to skip exon 51. A. The 1D amplitude plot shows positive (blue) and negative dots (grey) for the skipped and the non-skipped assays. B. Results of the electrophoresis run of the high sensitivity DNA assay showing the non-skipped fragment at 539 bp and the skipped fragment at 306 bp. C. The agarose gel shows the two fragments after electrophoresis; the non-skipped fragments at 475 bp and the skipped fragment at 242 bp. (TIF) [file pone.0204485.s001.tif]

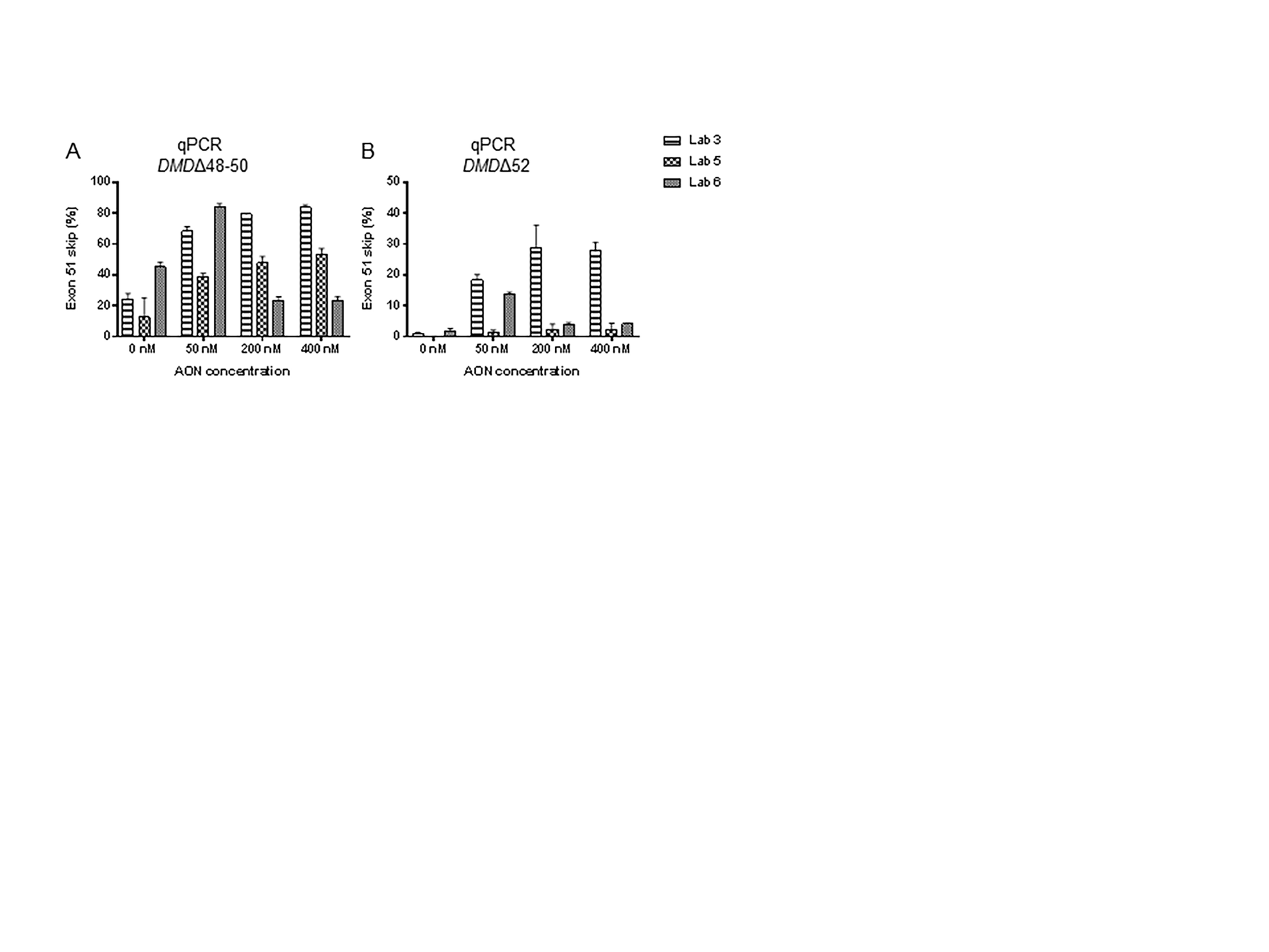

Supplement: S2 Fig — Intra-laboratory variation of exon 51 skipping levels in DMDΔ48–50 (A-C) and DMDΔ52 cells (D-E). Three different protocols were repeated by the same operator (n = 2/3). Error bars represent standard deviation. (TIF) [file pone.0204485.s002.tif]

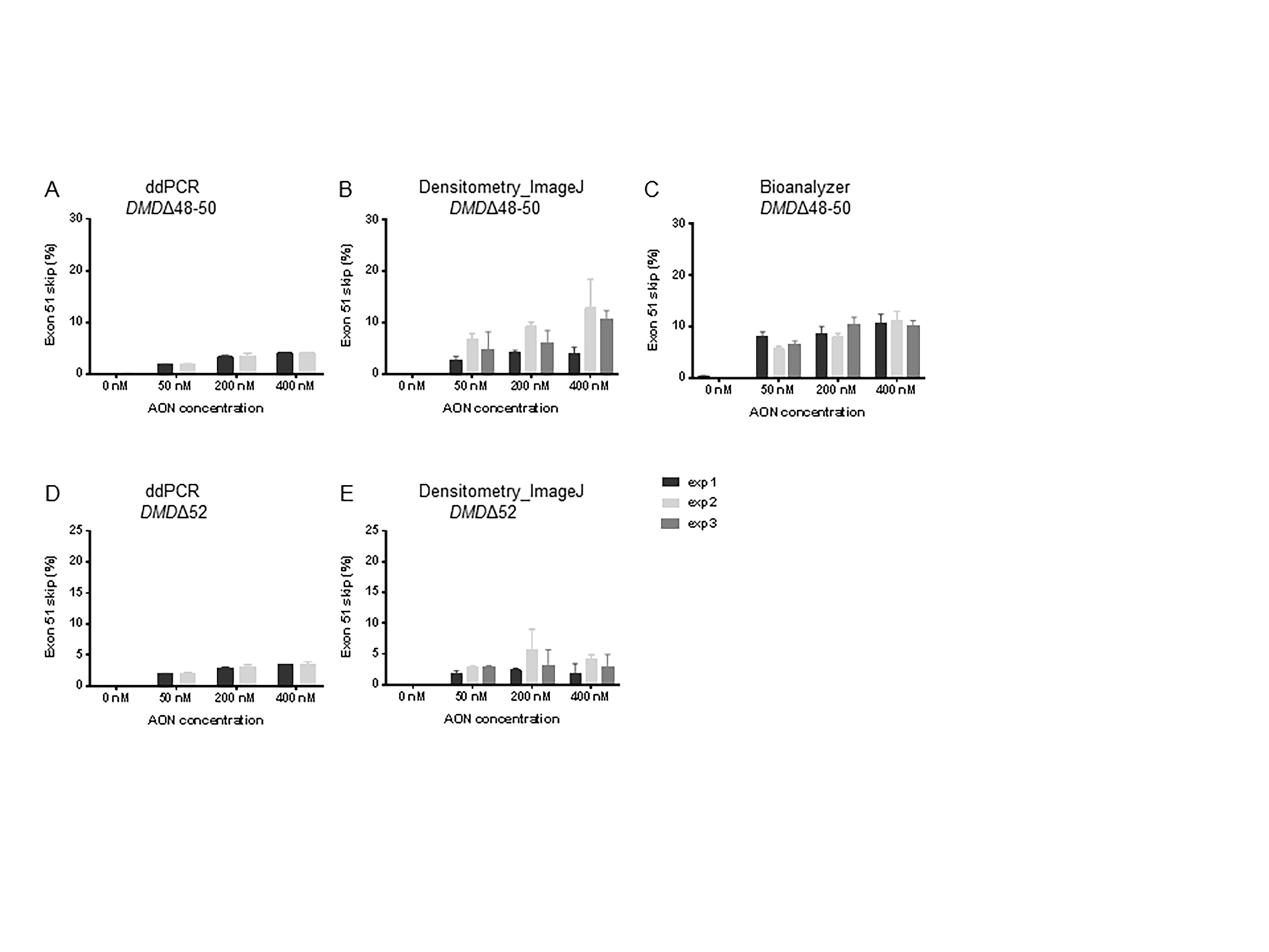

Supplement: S3 Fig — Error bars represent standard deviation. (TIF) [file pone.0204485.s003.tif]
