# Supplementary material for: A multicenter comparison of quantification methods for antisense oligonucleotide-induced DMD exon 51 skipping in Duchenne muscular dystrophy cell cultures
Source: PLoS One. 2018 Oct 2;13(10):e0204485. doi: 10.1371/journal.pone.0204485 (PMC6168132; doi:10.1371/journal.pone.0204485)
Supplement: S1 Table — FAM = 6-carboxyfluorescein label, 2’OMePS = 2'-O-methyl-modified bases on a phosphorothioate backbone. (DOCX) [file pone.0204485.s004.docx]

| **Oligonucleotide** | **Name** | ***DMD* deletion** | **Sequence (5’🡪 3’)** | **Target exon** | **Protocol** |
| --- | --- | --- | --- | --- | --- |
| AON (2'OMePS) | h51AON2 | Δ48-50, Δ52 | FAM - CCUCUGUGAUUUUAUAACUUGAU | 51 | 1^st^ and 2^nd^ transfection experiment |
| Primer | Skip_del48-50 | Δ48-50 | TGAAAATAAGCTCAAGCAGACAAATC | 47 | ddPCR method [30] |
| Primer |  |  | GACGCCTCTGTTCCAAATCC | 52 |  |
| Probe |  |  | FAM - CAGTGGATAAAGGCAACA | 47/52 |  |
| Primer | Non-skip_del48-50 | Δ48-50 | GTGATGGTGGGTGACCTTGAG | 51 | ddPCR method [30] |
| Primer |  |  | GACGCCTCTGTTCCAAATCC | 52 |  |
| Probe |  |  | FAM – CAAGCAGAAGGCAACAA | 51/52 |  |
| Primer | Skip_del52 | Δ52 | CCTGACCTAGCTCCTGGACTGA | 50 | ddPCR method [30] |
| Primer |  |  | TGTACTTCATCCCACTGATTCTGAA | 53 |  |
| Probe |  |  | FAM – CTATTGGAGCCTTTGAAAG | 50/53 |  |
| Primer | Non-skip_del52 | Δ52 | GTGACCTTGAGGATATCAACGAGAT | 51 | ddPCR method [30] |
| Primer |  |  | TGTACTTCATCCCACTGATTCTGAA | 53 |  |
| Probe |  |  | FAM – ATCAAGCAGAAGTTGAAAG | 51/53 |  |
| Primer | h46F | Δ48-50 | GTTTTATGGTTGGAGGAAGCAGAT | 46 | Bioanalyzer method |
| Primer | h52R | Δ48-50 | TTTGGGCAGCGGTAATGAG | 52 | Bioanalyzer method |
| Primer | h49F | Δ52 | CGGATGTGGAAGAGATTTTGTCT | 49 | Bioanalyzer method |
| Primer | h53R1 | Δ52 | GGATTGCATCTACTGTATAGGGAC | 53 | Bioanalyzer method |
| Primer | h47F1 | Δ48-50, Δ52 | TGAAACTGGAGGACCCGTG | 47 | Densitometry methods |
| Primer | h54R | Δ48-50, Δ52 | CCAAGAGGCATTGATATTCTC | 54 | Densitometry methods |
| Primer | h47F2 | Δ48-50, Δ52 | CCCATAAGCCCAGAAGAGC | 47 | Densitometry methods |
| Primer | h53R2 | Δ48-50, Δ52 | CTCCGGTTCTGAAGGTGTTC | 53 | Densitometry methods |
| Primer | Non-skip_ exon51-52 | Δ48-50 | GTGATGGTGGGTGACCTTGAG | 51 | qPCR method [31] |
| Primer |  |  | TTTGGGCAGCGGTAATGAG | 52 |  |
| Probe |  |  | FAM - CAAGCAGAAGGCAACAA | 51/52 |  |
| Primer | Skip_exon47-52 | Δ48-50 | TGAAAATAAGCTCAAGCAGACAAATC | 47 | qPCR method [31] |
| Primer |  |  | GACGCCTCTGTTCCAAAATCC | 52 |  |
| Probe |  |  | FAM - CAGTGGATAAAGGCAACA | 47/52 |  |
| Primer | Non-skip_exon51-53 | Δ52 | CCTTGAGGATATCAACGAGATGATC | 51 | qPCR method [31] |
| Primer |  |  | TTCTTGTACTTCATCCCACTGATTCT | 53 |  |
| Probe |  |  | FAM - TCAAGCAGAAGTTGAAAGA | 51/53 |  |
| Primer | Skip_exon50-53 | Δ52 | CCTGACCTAGCTCCTGGACTGA | 50 | qPCR method [31] |
| Primer |  |  | TTCTTGTACTTCATCCCACTGATTCT | 53 |  |
| Probe |  |  | FAM - ATTGGAGCCTTTGAAAGA | 50/53 |  |
