# Supplementary material for: A multicenter comparison of quantification methods for antisense oligonucleotide-induced DMD exon 51 skipping in Duchenne muscular dystrophy cell cultures
Source: PLoS One. 2018 Oct 2;13(10):e0204485. doi: 10.1371/journal.pone.0204485 (PMC6168132; doi:10.1371/journal.pone.0204485)
Supplement: S2 Table — Three biological replicates of each sample were measured. SD = standard deviation. (DOCX) [file pone.0204485.s005.docx]

| **AON concentrations** | | | | | | | | **Technology** | **Performing lab** | **Set of sample** |
| --- | --- | --- | --- | --- | --- | --- | --- | --- | --- | --- |
| **0 nM** | | **50 nM** | | **200 nM** | | **400 nM** | |  |  |  |
| **Exon 51 skip (%)** | **SD** | **Exon 51 skip (%)** | **SD** | **Exon 51 skip (%)** | **SD** | **Exon 51 skip (%)** | **SD** |  |  |  |
| 0.10 | 0.01 | 1.97 | 0.12 | 2.93 | 0.15 | 3.43 | 0.06 | ddPCR | lab 2 | 1st transfection *DMD*Δ52 |
| 0.09 | 0.01 | 1.93 | 0.24 | 2.75 | 0.14 | 3.34 | 0.38 | ddPCR | lab 3 | 1st transfection *DMD*Δ52 |
| 0.00 | 0.00 | 1.73 | 0.55 | 2.33 | 0.32 | 1.83 | 1.59 | Densitometry_ImageJ | lab 3 | 1st transfection *DMD*Δ52 |
| 0.00 | 0.00 | 5.63 | 2.85 | 10.10 | 13.17 | 6.35 | 0.78 | Densitometry_ImageJ | lab 4 | 1st transfection *DMD*Δ52 |
| 0.00 | 0.00 | 2.73 | 1.07 | 4.30 | 3.77 | 4.67 | 0.38 | Densitometry_ImageJ | lab 5 | 1st transfection *DMD*Δ52 |
| 0.00 | 0.00 | 4.27 | 1.72 | 6.80 | 2.36 | 5.67 | 2.48 | Densitometry_ImageJ | lab 6 | 1st transfection *DMD*Δ52 |
| 0.00 | 0.00 | 0.51 | 0.40 | 3.88 | 3.46 | 4.48 | 3.95 | Densitometry_GeneTools | lab 4 | 1st transfection *DMD*Δ52 |
| 0.30 | 0.14 | 6.43 | 1.33 | 6.90 | 0.95 | 7.57 | 1.62 | Bioanalyzer | lab 3 | 1st transfection *DMD*Δ52 |
| 0.03 | 0.06 | 6.61 | 1.15 | 7.36 | 0.41 | 8.19 | 0.60 | Bioanalyzer | lab 6 | 1st transfection *DMD*Δ52 |
| 1.11 | 0.27 | 18.36 | 1.78 | 28.96 | 7.20 | 27.73 | 2.85 | qPCR | lab 3 | 1st transfection *DMD*Δ52 |
| 0.10 | 0.07 | 1.38 | 0.81 | 2.26 | 1.90 | 2.16 | 2.18 | qPCR | lab 5 | 1st transfection *DMD*Δ52 |
| 1.88 | 0.80 | 13.82 | 0.67 | 4.02 | 0.62 | 4.21 | 0.26 | qPCR | lab 6 | 1st transfection *DMD*Δ52 |
| 0.16 | 0.01 | 1.93 | 0.06 | 3.30 | 0.30 | 4.07 | 0.15 | ddPCR | lab 2 | 1st transfection *DMD*Δ48-50 |
| 0.18 | 0.00 | 2.39 | 0.08 | 3.73 | 0.14 | 4.65 | 0.43 | ddPCR | lab 3 | 1st transfection *DMD*Δ48-50 |
| 0.00 | 0.00 | 2.67 | 0.75 | 4.27 | 0.35 | 3.83 | 1.33 | Densitometry_ImageJ | lab 3 | 1st transfection *DMD*Δ48-50 |
| 0.00 | 0.00 | 13.03 | 1.10 | 14.37 | 1.45 | 21.13 | 6.94 | Densitometry_ImageJ | lab 4 | 1st transfection *DMD*Δ48-50 |
| 0.00 | 0.00 | 4.40 | 1.15 | 8.00 | 3.94 | 14.17 | 8.75 | Densitometry_ImageJ | lab 5 | 1st transfection *DMD*Δ48-50 |
| 0.00 | 0.00 | 10.33 | 2.00 | 20.20 | 6.30 | 21.67 | 2.25 | Densitometry_ImageJ | lab 6 | 1st transfection *DMD*Δ48-50 |
| 0.00 | 0.00 | 2.05 | 0.38 | 2.47 | 0.34 | 3.27 | 1.15 | Densitometry_GeneTools | lab 4 | 1st transfection *DMD*Δ48-50 |
| 0.50 | 0.44 | 8.03 | 1.03 | 8.33 | 2.30 | 8.93 | 3.04 | Bioanalyzer | lab 3 | 1st transfection *DMD*Δ48-50 |
| 0.00 | 0.00 | 3.21 | 1.04 | 6.66 | 2.44 | 10.11 | 5.95 | Bioanalyzer | lab 4 | 1st transfection *DMD*Δ48-50 |
| 0.41 | 0.11 | 8.16 | 0.82 | 8.58 | 1.44 | 10.68 | 1.72 | Bioanalyzer | lab 6 | 1st transfection *DMD*Δ48-50 |
| 24.21 | 3.92 | 68.28 | 2.89 | 79.35 | 0.77 | 83.85 | 1.62 | qPCR | lab 3 | 1st transfection *DMD*Δ48-50 |
| 12.65 | 12.65 | 38.61 | 2.40 | 47.71 | 4.30 | 53.15 | 4.09 | qPCR | lab 5 | 1st transfection *DMD*Δ48-50 |
| 45.13 | 3.14 | 84.36 | 1.89 | 23.30 | 2.55 | 23.58 | 2.47 | qPCR | lab 6 | 1st transfection *DMD*Δ48-50 |
| 0.10 | 0.00 | - | - | 13.30 | 0.85 | 15.53 | 0.25 | ddPCR | lab 2 | 2nd transfection *DMD*Δ48-50 |
| 0.11 | 0.02 | - | - | 14.07 | 1.00 | 16.72 | 1.34 | ddPCR | lab 3 | 2nd transfection *DMD*Δ48-50 |
| 0.14 | 0.06 | - | - | 18.16 | 2.65 | 21.49 | 1.49 | Bioanalyzer | lab 1 | 2nd transfection *DMD*Δ48-50 |
| 0.40 | 0.17 | - | - | 28.37 | 1.33 | 34.17 | 1.23 | Bioanalyzer | lab 3 | 2nd transfection *DMD*Δ48-50 |
| 0.26 | 0.24 | - | - | 19.48 | 3.22 | 24.39 | 1.19 | Bioanalyzer | lab 4 | 2nd transfection *DMD*Δ48-50 |
| 0.51 | 0.02 | - | - | 24.48 | 1.72 | 26.60 | 2.34 | Bioanalyzer | lab 5 | 2nd transfection *DMD*Δ48-50 |
| 0.71 | 0.39 | - | - | 28.45 | 2.64 | 31.77 | 2.86 | Bioanalyzer | lab 6 | 2nd transfection *DMD*Δ48-50 |
