# Supplementary material for: A multicenter comparison of quantification methods for antisense oligonucleotide-induced DMD exon 51 skipping in Duchenne muscular dystrophy cell cultures
Source: PLoS One. 2018 Oct 2;13(10):e0204485. doi: 10.1371/journal.pone.0204485 (PMC6168132; doi:10.1371/journal.pone.0204485)
Supplement: S1 Supplementary information — (DOCX) [file pone.0204485.s006.docx]

**Supplementary information: Cell culturing, AON transfection and RNA isolation**

**First transfection experiment**

Primary human patient myoblasts with a deletion of exon 52 (*DMD*Δ52, cell line 1531) or exons 48-50 (*DMD*Δ48-50, cell line 8036) [27,28] were cultured in skeletal muscle cell growth medium (#C-23160, PromoCell) supplemented with 10% fetal bovine serum (#10270, Thermo Fisher Scientific), 1% glutamax (#35050, Thermo Fisher Scientific), 1% penicillin-streptomycin (PS, #15070, Thermo Fisher Scientific) and 5 µg/ml gentamicin (#P11-004, PAA Laboratories) in 0.5% gelatin (#G1890, Sigma-Aldrich) coated 6-well plates at 37˚C with 5% CO_2_ supply. After cells were 70–80% confluent, the medium was switched to differentiation medium containing Dulbecco's modified eagle medium (DMEM, #BE12-604F, Lonza), 2% horse serum (#26050, Thermo Fisher Scientific) and 1% PS (#15070, Thermo Fisher Scientific). Myotubes were transfected after one day on differentiation medium with AON h51AON2 (S1 Table, provided by BioMarin) [29] to skip exon 51 by using 2 μl of transfection reagent lipofectamine 2000 (#11668019, Thermo Fisher Scientific) according to manufacturer’s instructions in a total volume of 1 ml per well. AONs were transfected at 50, 200 and 400 nM to obtain low, medium and high exon skipping levels. Lipofectamine-only treated cells, “untreated cells” were used as a negative control. Per condition, two 6-well plates per cell line were included to have enough starting material for all experiments. After 5 hours, the medium was changed to differentiation medium and cells were further incubated at 37˚C prior to RNA isolation.

RNA was isolated 48h after transfection with the High Pure RNA Isolation Kit (#11828665001, Roche) according to the manufacturer’s instructions. Four wells of a 6-well plate were pooled together to create three replicates per condition. The RNA pellet was eluted in 80 μl of elution buffer from the kit and RNA samples were measured on a NanoDrop 1000 Spectrophotometer (Thermo Fisher Scientific) to determine RNA concentration and purity. Exon skipping was confirmed prior to delivery to the participating partners.

**Second transfection experiment**

Cell culturing, transfection and RNA isolation were performed with minor variations for the second transfection experiment. In this second experiment, at another laboratory, *DMD*Δ48-50 cells (cell line 8036) were grown in skeletal muscle cell growth medium (#C-23160, PromoCell) supplemented with 15% fetal bovine serum (#10270, Thermo Fisher Scientific) and 50 µg/ml gentamicin (#P11-004, PAA Laboratories) in 0.5% gelatin (#G1890, Sigma-Aldrich) coated 6-well plates at 37˚C with 5% CO_2_ supply. After cells reached 70–80% confluency, the medium was switched to differentiation medium containing Dulbecco’s medium without phenol red (#11880028, Thermo Fisher Scientific), 2% fetal bovine serum, 50 µg/ml gentamicin, 2% glutamax (#35050038, Thermo Fisher Scientific) and 1% glucose (#G8769, Sigma-Aldrich). Within 4 days, myoblasts had differentiated into myotubes to be transfected with AON h51AON2 (S1 Table, provided by BioMarin) [29]. One 6-well plate per AON condition was used to transfect myotubes either with 200 or 400 nM of AON. Untreated cells were used as a negative control. The transfections were performed with 6 ul of lipofectamine 2000 reagent (#11668019, Thermo Fisher Scientific) according to the manufacturer’s instructions in a total volume of 2 ml per well. Three hours after the transfection, medium was changed to differentiation medium and cells were further incubated at 37˚C prior to RNA isolation.

RNA was isolated with TriPure reagent from Roche (#11667165001) according to manufacturer’s instructions 48h after transfection. RNA obtained from two wells of a 6-well plate of the same condition were pooled together to create three replicates per AON condition. The RNA pellet was suspended in 22 μl RNase/DNase-free water and RNA concentrations were measured on a NanoDrop 1000 Spectrophotometer. Exon skipping levels were performed according to the protocol ‘Single PCR combined with Agilent bioanalyzer analysis’ (see Materials and Methods section) before samples were shipped to the participating partners.
